# Tribological Properties of Ti6Al4V Alloy Composite Texture Fabricated by Ultrasonic Strengthening Grinding and Laser Processing

**DOI:** 10.3390/ma16010355

**Published:** 2022-12-30

**Authors:** Jinrui Xiao, Yiteng Zhang, Bin Hu, Xiaochu Liu, Zhongwei Liang, Zhuan Zhao

**Affiliations:** 1 Guangdong Engineering Research Centre for Strengthen Grinding and Micro\Nano High-Performance Machining, Guangzhou University, Guangzhou 510006, China; 2 School of Mechanical and Electrical Engineering, Guangzhou University, Guangzhou 510006, China; 3 School of Physics and Materials Science, Guangzhou University, Guangzhou 510006, China

**Keywords:** Ti6Al4V alloy, ultrasonic strengthening grinding process, laser texture, wear resistance

## Abstract

The Ti6Al4V alloy has been widely used in aerospace equipment and medical devices. However, the poor wear resistance of the Ti6Al4V alloy hinders its further engineering application. In this study, the ultrasonic strengthening grinding process (USGP) and laser texturing process were employed to enhance the wear resistance of Ti6Al4V alloy. The frictional behavior of all samples was determined via a ball-on-disc friction and wear tester under dry conditions. The worn surface morphology, cross-sectional hardness, surface roughness, and microstructure were analyzed. The results demonstrated that the USGP induced high hardness, high dislocation density, and grain refinement, as well as improvements in the wear resistance of Ti6Al4V. Moreover, laser texture could enhance the capacity to capture wear debris and reduce wear probability. When combining the USGP and laser texturing process for the surface treatment of Ti6Al4V alloy, the lowest and most stable friction coefficients were obtained, as well as the best wear resistance. Compared to the polished sample, the steady stage friction coefficient of the sample treated by USGP and laser texturing process was remarkably decreased by 58%. This work demonstrates that combining the USGP and laser texturing process could be a promising solution for improving the wear resistance properties of Ti6Al4V alloy, which makes it more suitable for various engineering applications.

## 1. Introduction

Ti6Al4V is widely applied in aerospace equipment, the automotive industry, and medical devices owing to its good corrosion resistance, low density, high strength, and fracture toughness [1,2,3]. However, it is confronted with the inevitable challenges of poor wear resistance [4]. These shortcomings have severely hindered the Ti6Al4V alloy from being used on a larger scale for various applications [5,6]. Thus, a strengthening process must be conducted to improve the wear resistance properties of Ti6Al4V alloy.

To address this issue, many solutions have been proposed, such as surface coating, heat treatment, rolling, shot peening, and surface texturing [7,8,9,10,11]. As one of the most commonly used methods, heat treatment can introduce grain refinement, high hardness, high strength, and excellent mechanical properties. Huang et al. [12] found that TiC particles might prevent the growth of the matrix during the quenching process. Furthermore, the evidence of phase change and hardness improvement induced by heat treatment was proved by Jeong et al. [13]. However, some disadvantages, such as high-temperature deformation and high energy consumption, that occur during heat treatment are inevitable. It is well known that preparing a hard coating on the contacted surface is an effective solution to overcome the shortcoming of the poor wear resistance of Ti6Al4V alloy. Liu et al. [14] found that higher microhardness, lower friction coefficient, and lower wear rate could be obtained via fabricating a compositionally graded Ti-Al intermetallic coating on the surface of Ti6Al4V alloy. Panjwani et al. [15] also proved that a remarkable increase in wear resistance could be obtained when a wear-resistant coating was fabricated on the surface of titanium alloy. The previous studies showed that residual stress and porous were inevitably introduced, which might impact coating adherence and subsequent performance, during coating processing [16]. To tackle these issues, a promising solution that combines heat treatment and a surface coating process was proven [17]. For example, Chen et al. [18] prepared TiC/TiB composite bioinert ceramic coatings via laser cladding and decreased the negative effect of the excessive residual tensile stress by conducting heat treatment. As a result, an excellent wear resistance property was subsequently obtained. Nevertheless, the coupling technical parameters need to be controlled carefully, since the consistency of material properties is challenging to guarantee during processing. This issue will further have adverse effects on wear resistance and practical application.

As an alternative, rolling processing offers a new approach to producing a texture or strengthening layer, which is believed to be beneficial in improving the strength and wear resistance of the metallic material surface [19,20]. However, it may induce severe deformation or lead to profound structural changes [21,22]. On the other hand, shot peening is an efficient method of achieving good tribological properties on contacted surfaces [23]. Yan et al. found that the microhardness and wear resistance of samples treated by shot peening were significantly increased [24]. Similar results were obtained by Liu et al., owing to the grain refinement and compressive residual stress enhancement introduced by shot peening [25]. However, the high surface roughness may be induced during shot peening processing [26]. In the past decade, surface texturing has emerged as a promising method for improving the tribological properties of contacted surfaces [27,28,29]. Surface texture is proven to have the extra ability to capture worn abrasives, reduce contact area, and provide secondary lubrication [30,31]. Thus, excellent load capacity, wear resistance, and tribological properties can be obtained when the surface is textured. Nevertheless, the textured surface layer cannot be enhanced during conventional texturing processing, such as layer processing or electrochemical machining [32]. Moreover, severe oxidation and ablation are inevitably induced, which affects the strength and hardness of the treated surface [33]. When the strength and hardness of the matrix are poor, the texture will be easily worn in the following practical application. Hence, it is necessary to enhance the strength of the texture matrix.

On the other hand, the ultrasonic strengthening grinding process (USGP) offers an efficient solution that impacts the target samples with a mixed abrasive of brown corundum, fused ceramic balls, and strengthening liquid, to enhance the wear resistance [34,35]. Liu et al. [36] investigated the influence of mixed abrasive impacting on the tribological properties of 30CrMnSiA bearing steels and found that the friction coefficient and wear volume were decreased. Xiao et al. [35] discovered that the average friction coefficient of samples treated with USGP was significantly lower than that of untreated samples. Thus, the USGP shows perfect universality for enhancing the wear resistance of metallic materials. However, although a strengthened surface layer with texture can be obtained via the USGP process, its texture shape is irregular and the depth is shallow, which is not conducive to storing a large amount of wear debris [36].

To address this issue, the present research combines the advantages of texturing and USGP for improving the wear resistance of Ti6Al4V alloy. For the first time, a comprehensive comparison between the Ti6Al4V alloy samples fabricated by USGP or layer texturing on their friction coefficient, worn surface morphology, and wear characteristics has been carried out in the current study. Most importantly, this work provides a fundamental understanding of its tribological properties enhancement by evaluating the evolutions of surface micromorphology, microstructure, and hardness.

## 2. Materials and Methods

### 2.1. Materials

**Figure 1:**
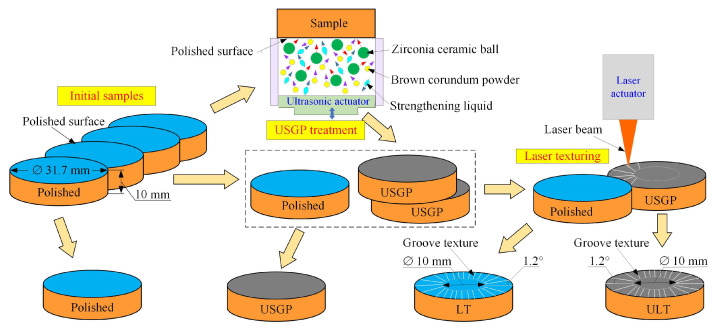
The geometrical parameters of samples.

A commercial Ti6Al4V titanium alloy with the chemical composition illustrated in Table 1 was used in the current study. The Ti6Al4V alloy was annealed at 765 °C for 2.5 h and cooled in the air to achieve an average surface hardness of 321.3 HV. Four discs with a diameter of 31.7 mm and a thickness of 10 mm were prepared, as shown in Figure 1. All the upper and lower surfaces were polished with emery paper of 800 grit, resulting in an average surface roughness of *Ra* below 0.33 µm, and then cleaned with alcohol in an ultrasonic bath. 

### 2.2. Surface Strengthening and Texture Fabrication

**Figure 2:**
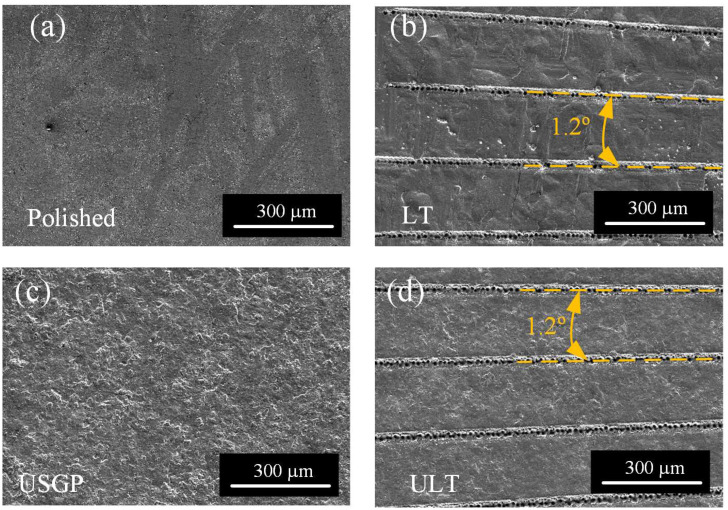
SEM topography of polished (**a**), LT (**b**), USGP (**c**), and ULT (**d**) samples.

Two samples experienced the process of the USGP, which was illustrated in our previous work [35]. As shown in Figure 1, the mixed abrasives used for the USGP are made up of zirconia ceramic balls (1.5 mm), brown corundum powder (15 μm), and strengthening liquid. The ultrasonic vibration frequency was fixed at 20 kHz, and the processing time was set at 300 s. Finally, two samples were treated by the USGP. A nanosecond laser (Q1, Aunion Tech Co., Ltd., Shanghai, China) was employed for the texture fabrication of the samples. The processing parameters are given in Table 2. The groove textures were fabricated, respectively, on the surfaces of two samples that underwent different treatments. Texture processing was conducted in the ambient air at 25 °C. The length of each groove was 10.85 mm. The angle between the centers of two adjacent linear grooves was 1.2°, which resulted in 300 microgrooves on each textured surface. Finally, four samples were prepared (see Figure 1), and the surface topography is shown in Figure 2. The samples’ surfaces treated by polishing, USGP, laser texturing, and USGP followed by laser texturing are named polished, USGP, LT, and USLT, respectively.

### 2.3. Materials Characterization

Four samples with geometrical parameters of 10 mm × 8 mm × 5 mm were cut off from the previous discs. The microhardness tests were performed on the cross-section of all samples using a Vickers hardness tester (HV-1000, Shanghai optical instrument factory, Shanghai, China). The cross-sections perpendicular to the treated surface were ground off with a thickness of about 0.5 mm, followed by a polishing procedure with grinding paper (800 grit) and diamond paste (2.5 μm) to ensure the roughness of *Ra* was below 0.5 μm. After that, all samples were cleaned with alcohol in an ultrasonic bath. The cross-sectional microhardness from the treated surface to a depth of 400 μm, with a step of 20 μm, was measured by a Vickers hardness tester. A loading force of 0.3 N and held for 10 s was fixed. Five points were tested at each depth, and the final microhardness was obtained from the average value. 

A scanning electron microscope (SEM; JSM-7001F, JEOL Ltd., Tokyo, Japan) was used to characterize the micromorphology of the treated surface. The cross-sectional microstructure was observed by optical microscopy (OM, MJ-42, Mshot Photoelectric Ltd., Guangzhou, China). An interferometer optical microscope (Contour GT-K 3D, Bruker Inc., Karlsruhe, Germany) was utilized to obtain surface topography and roughness. 

The phase compositions of the polished, USGP, LT, and USLT samples were identified via an X-ray diffractometer (Rigaku +Ultima IV, Rigaku Corp., Akishima, Tokyo, Japan). The measured surfaces were detected by Cu-Kα in a scanning angle range of 30° to 90° with an interval step of 1° under 30 mA at 40 kV. 

### 2.4. Tribological Tests

**Figure 3:**
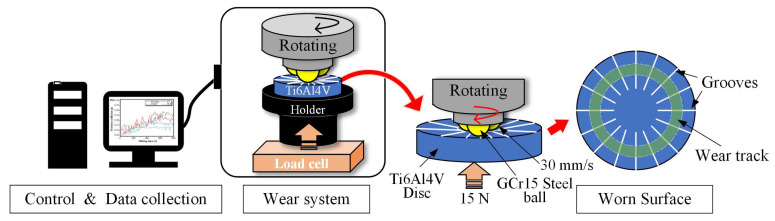
Schematic diagram of the ball-on-disc tribological test system.

The tribological tests were carried out on the ball-on-disc rotating friction and wear tester (MVF-2A, Jinan Hengxu Testing Machine Technology Inc., Jinan, China), the schematic diagram of which is shown in Figure 3. GCr15 steel balls with a diameter of 6.35 mm and a hardness of 700 ± 20 HV were conducted as the upper grinding piece. A normal load of 15 N, an average sling velocity of 30 mm/s, and a total sliding time of 3600 s were set during the tribological tests. All tests were carried out in conditions of 50 % ambient humidity and 25 °C temperature. The samples were ultrasonically cleaned with alcohol for 15 min before tribological tests. The friction coefficient in the sliding tests was measured continuously. After tribological tests, the wear tracks and chemical compositions were detected by a JSM-7001F SEM equipped with an energy dispersive spectrometer (EDS) detector. Moreover, a Contour GT-K 3D interferometer optical microscope was utilized to measure the worn surface microtopography, wear track cross-sectional profile, and wear volume.

## 3. Results and Discussion

### 3.1. Hardness and Roughness Analysis

**Figure 4:**
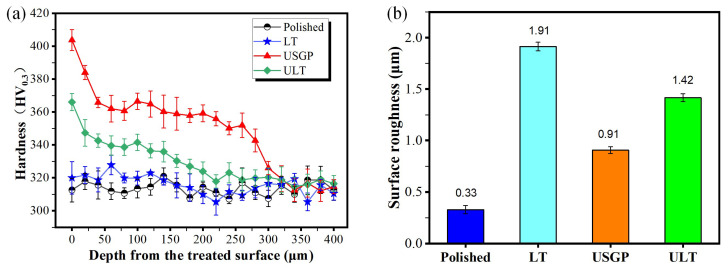
The variation hardness of cross-section (**a**) and the treated surface roughness *Ra* (**b**) of each sample.

The experimental results for the average hardness are shown in Figure 4a. The average hardness of the polished sample is about 317 HV_0.3_. A significant increase in hardness is found in the USGP and ULT samples. The USGP sample has the maximum hardness from the treated surface to the matrix among these samples. Moreover, a maximum average hardness of 403.7 HV_0.3_ is obtained at the top surface of the USGP sample, which presents an increase of 10.4%, 26.3%, and 27.4% compared with the ULT, LT, and polished samples, respectively. The hardness of samples USGP and ULT decreases with the increase of depth and drops to 318 HV_0.3_ at a depth of 320 μm and 220 μm, which is close to that of the polished sample. Hence, the hardened layers of the USGP and ULT samples are about 320 μm and 220 μm, respectively. The USGP treatment has been proven to induce work hardening, while laser texturing may introduce high temperatures and anneal on the treated surface [34,37]. Thus, the hardness of the strengthening layer will decrease when it undergoes surface texturing. As a result, the hardness of the ULT sample is slightly lower than that of the USGP sample. 

The arithmetic mean roughness of the samples’ surface was characterized, as shown in Figure 4b. A minimum surface roughness of 0.33 μm is obtained in the polished sample, which is 17.28%, 36.26%, and 23.24% of the LT, USGP, and ULT samples, respectively. Obviously, the laser texture process and USGP treatment would also increase the surface roughness. In the laser texturing process, oxidized debris and edge bulge form, leading to a significant increase in the surface roughness of the samples. 

### 3.2. Microstructure Analysis of Samples

**Figure 5:**
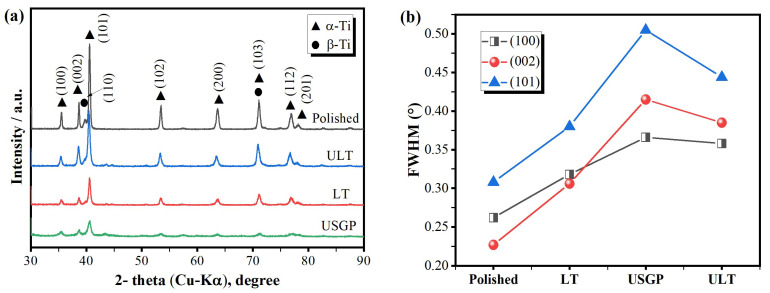
X-ray diffraction pattern (**a**), the variation of FWHM of α (100), (002), and (101) peaks (**b**) of overall treated samples before sliding wear.

The phase composition and dislocation density on the treated surface before the sliding wear test were studied through XRD analysis (see Figure 5). The XRD diffraction patterns of overall samples before the sliding wear test are illustrated in Figure 5a. The broadening of diffracted peaks appears in the ULT, LT, and USGP samples with the reduction of diffracted intensity. The widest diffracted peak is found in the USGP sample, which is attributed to the grain refinement and high-density dislocations induced by the high-velocity impact of the mixed abrasives [35,38]. When laser texturing was conducted, the diffracted intensity was reduced while the broadening of the diffracted peaks occurred. This phenomenon indicated that grain refinement might also occur during the laser texturing process. As reported in Figure 5b, the full width at half maximum (FWHM) of the USGP sample has the most significant value. The FWHM of the α (101) peaks of the USGP sample is 0.505º, which is increasing by 64.0%, 32.9%, and 13.7%, respectively, compared to the polished, LT, and ULT samples. Thus, high-density dislocation and grain refinement were proved to be introduced by the USGP treatment again [39]. 

**Figure 6:**
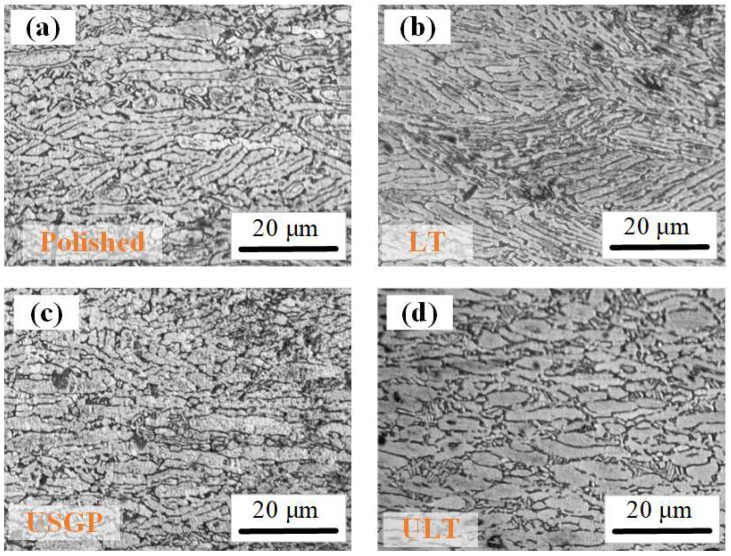
Cross-sectional microstructures of polished (**a**), LT (**b**), USGP (**c**), and ULT (**d**) samples.

Figure 6 illustrates the cross-sectional microstructures of samples treated via different processes. The elongated grains are arranged regularly in the cross-section of the polished sample, while a finer grain is observed in the LT sample (see Figure 6a,b). When USGP treatment was conducted, significant grain refinement was found in the USGP sample (see Figure 6c). However, grain coarsening occurred when USGP treatment was employed, followed by the laser texturing process, as shown in Figure 6d. High-temperature and high-energy impact might be introduced during the laser texturing process. Thus, grain refinement, coarsening, and microstructure deformation will then occur. Owing to the high-energy shock of the mixed abrasives, plastic deformation, dislocation, and strain will be induced in the treated surface layer. Therefore, grain refinement is observed in the cross-section of the USGP sample.

**Figure 7:**
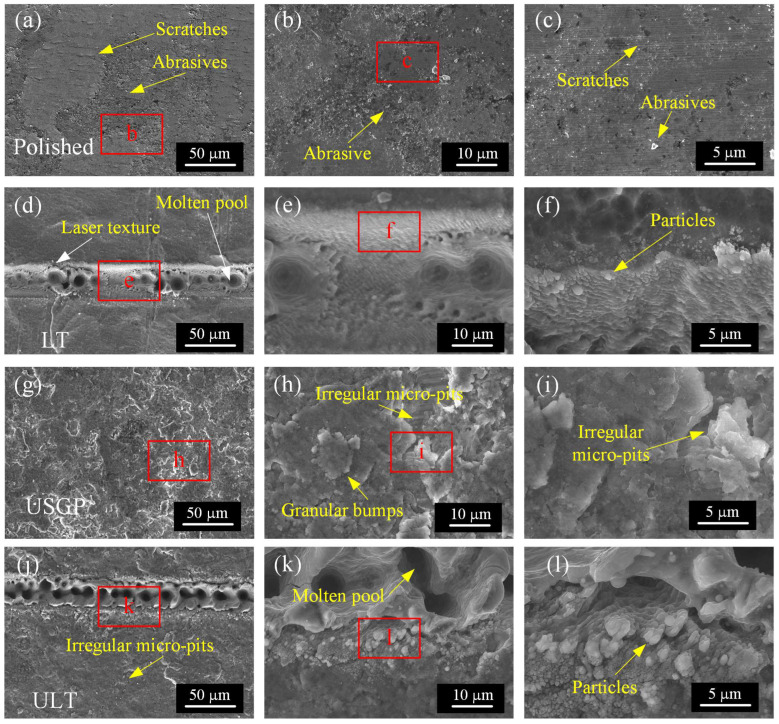
SEM images of the details of polished (**a**–**c**), LT (**d**–**f**), USGP (**g**–**i**), and ULT (**j**–**l**) samples before sliding wear. The figures (**b**,**e**,**h**,**k**) are the enlarged images of the areas with red letters in figure (**a**,**d**,**g**,**j**), and figure (**c**,**f**,**i**,**l**) are the enlarged appearances of the areas with red letters in figure (**b**,**e**,**h**,**k**), respectively.

Figure 7 displays the micrograph details of polished, LT, USGP, and ULT samples. The slight scratches and abrasives, which are introduced during the polishing processing, on the surface of the polished sample are visible, as shown in Figure 7a–c. The laser texture of grooves with molten pool and particles is found in the LT sample (Figure 7d–f). As contrasted with the polished and LT samples, irregular micro-pits and granular bumps can be observed on the surface of USPG (Figure 7g–i). Compared to the LT sample, some bigger particles and more uniform molten pools can be found on the surface of the ULT sample, as illustrated in Figure 7j–l. It has been reported in our previous works that the irregular micro–pits and granular bumps are contributed to the high-velocity shock of mixed abrasives during USGP treatment [35,36]. 

### 3.3. Tribological Properties

The detailed effect of polishing, laser texturing, USGP, and USGP followed by laser texturing on the tribological behavior of Ti6Al4V disc against GCr15 steel ball, is carried out in this section. 

**Figure 8:**
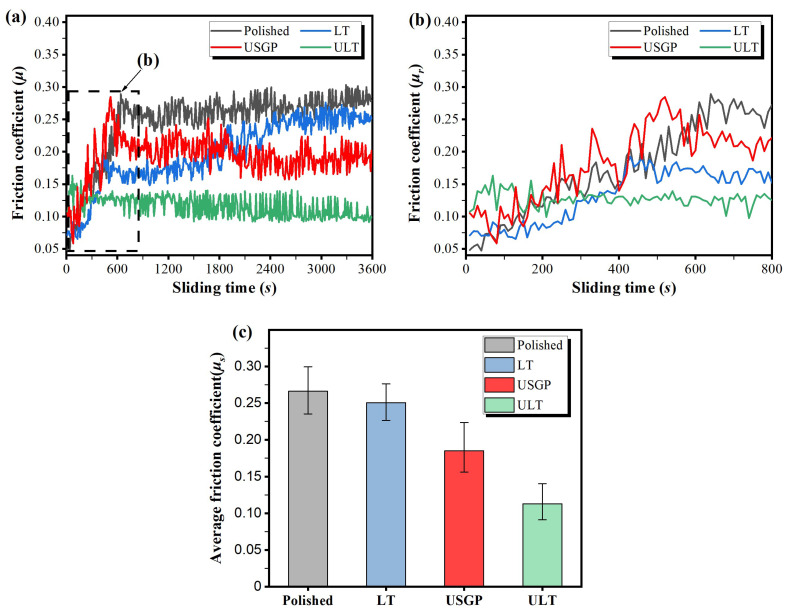
Variation in friction coefficient (*μ*) against the sliding time under a fixed normal load of 15 N at a constant sliding velocity of 30 mm/s for Ti6Al4V discs after different treatments (**a**); Variation in running-in state friction coefficient (*μ_r_*) against the sliding time (**b**) and the average friction coefficient (*μ_s_*) in steady-state of both samples (**c**).

Figure 8a presents the friction coefficient (*μ*) against sliding time under a load of 15 N at a constant sliding velocity of 30 mm/s with a total sliding time of 3600 s for Ti6Al4V discs with different treatments. The curves reveal that the friction coefficient strongly depends on the treatment method. Generally, the sample treated via USGP followed by laser texturing has a lower friction coefficient with fewer fluctuations than the others. During the whole sliding time, the *μ* of the LT sample can be divided into three stages, which are the rapid running-in stage, slightly increasing stage, and steady-state. A similar variation in *μ* of polished and USGP samples can be found. Figure 8b exhibits the friction coefficient (*μ_r_*) during the running-in state. In the first 50 s, the *μ_r_* of the polished sample is the lowest among the four. After that, the same increasing trends in *μ_r_* can be found in the polished, LT, and USGP samples when the sliding time is less than 450 s. A significantly different variation of the *μ_r_* is shown in the ULT sample, which fluctuates around 0.16 and 0.11 in the first 200 s and then goes to a steady state directly. Figure 8c summarizes the average friction coefficient (*μ_s_*) in a steady state. The lowest *μ_s_* of 0.113 is obtained in the ULT sample. It drops by about 39%, 55%, and 58%, respectively, compared to USGP, LT, and polished samples.

**Figure 9:**
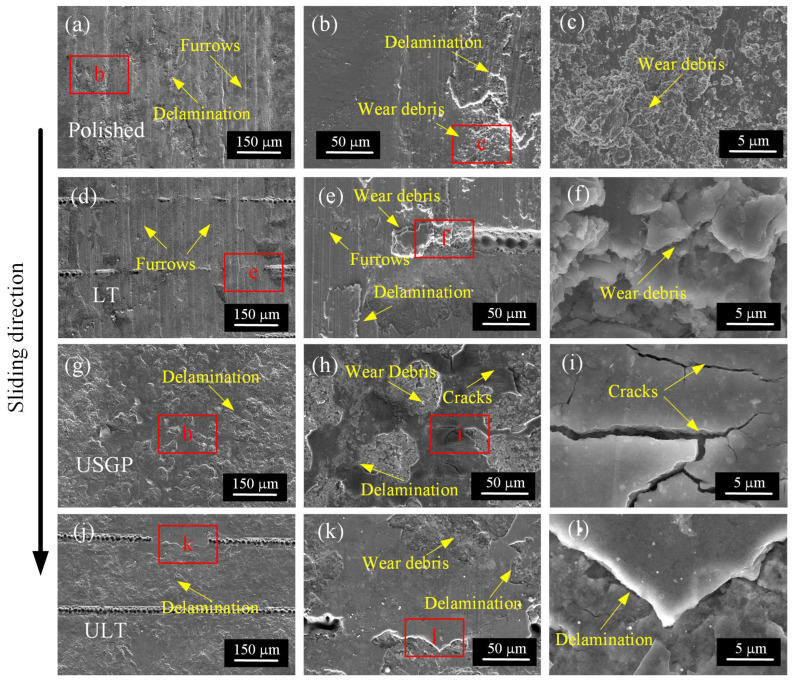
The worn surface morphologies of the polished (**a**–**c**), LT (**d**–**f**), USGP (**g**–**i**), and ULT (**j**–**l**) samples after sliding under a fixed normal load of 15 N for 3600 s at a constant sliding velocity of 30 mm/s. The figures (**b**,**e**,**h**,**k**) are the enlarged images of the areas with red letters in figure (**a**,**d**,**g**,**j**), and figures (**c**,**f**,**i**,**l**) are the enlarged appearances of the areas with red letters in figure (**b**,**e**,**h**,**k**), respectively.

Figure 9 illustrates the worn surface morphologies of polished, LT, USGP, and ULT samples after sliding at 30 mm/s under a constant normal load of 15 N. Many wear furrows along the sliding direction, accompanied by delamination and wear debris, appear on the worn surfaces (see Figure 9a–f). Indeed, finer wear debris is exhibited on the worn surface of the polished sample compared to the LT sample. When the USGP treatment is conducted for the polished surface or laser texturing is performed after USGP, the furrows disappear while cracks and delamination occur on the worn surface (see Figure 9g–l). Obviously, localized fatigue delamination is detected on the worn surface of the USGP and ULT samples. Moreover, delamination accompanied by no cracks can be found on the worn surface of the ULT sample, as evident from the magnified SEM images in Figure 9j–l. According to the above analysis, a conclusion can be drawn that the main characteristics are strongly related to the post-processing method. Furthermore, the USGP treatment is found to be an effective solution for addressing the abrasive scratches, while laser texturing figures out the wear debris. 

**Figure 10:**
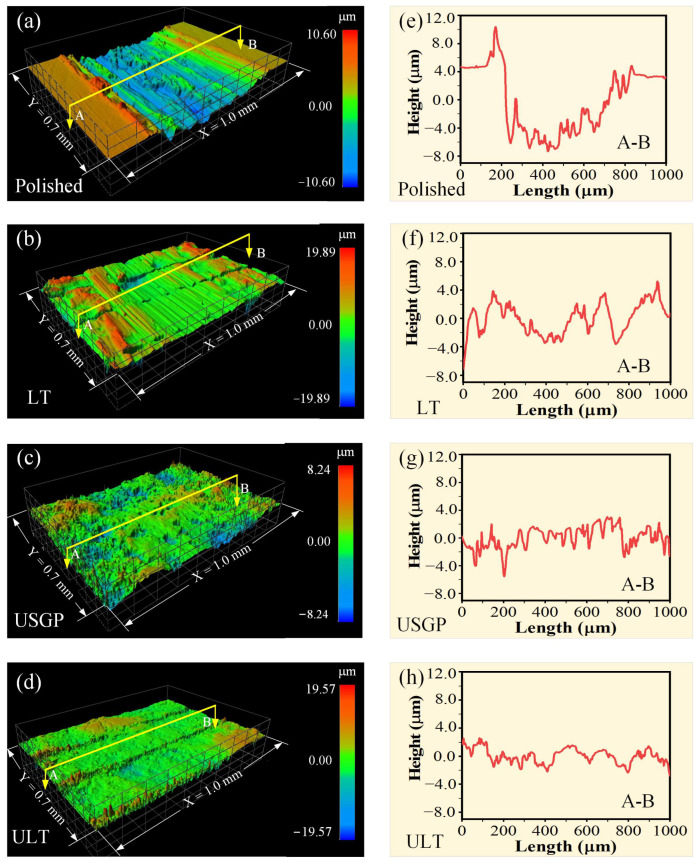
The micromorphology and cross-sectional profiles that took in the middle of the wear tracks of the polished (**a**,**e**), LT (**b**,**f**), USGP (**c**,**g**), and ULT samples (**d**,**h**).

Figure 10 presents the micromorphology and cross-sectional profiles of the wear tracks after sliding for 3600 s. Obviously, a deep (~12 μm) and wide (~700 μm) track with scratches and wear debris appear on the worn surface of the polished sample. A slight material accumulation on the track flanks and significant volume loss in the center of the wear track can be found. Only furrows exist on the scratch of the LT sample. Additionally, the wear debris was captured by the grooves fabricated via the laser texturing process. A total wide and maximum deep of wear track in 545 μm and 7 μm, which decrease by 22.14% and 41.67% compared to the polished sample, are observed in the LT sample. In the USGP sample, a scratch with a depth and width of ~6 µm and ~475 µm, respectively, is observed. In contrast, slightly abrasive and adhesive wear is found in the ULT sample, which decreases 66.08%, 41.86%, and 33.3%, respectively, in-depth compared to the polished, LT, and USGP samples. Therefore, the surface treatment of laser texturing performed after USGP treatment is proven again to be an efficient method for enhancing the wear resistance of Ti6Al4V alloy.

**Figure 11:**
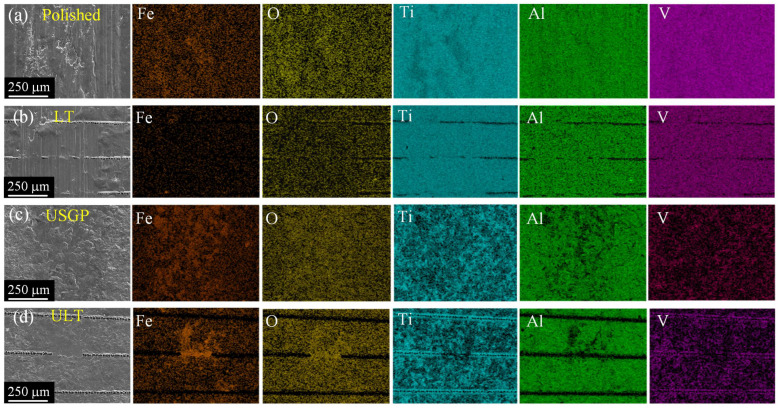
EDS elemental maps of the polished (**a**), LT (**b**), USGP (**c**), and ULT (**d**) samples after sliding under a fixed normal load of 15 N for 3600 s at a constant sliding velocity of 30 mm/s.

During the friction and wear test, a GCr15 steel ball slides against a Ti6Al4V alloy disc. Alternating sliding with loads of GCr15 steel balls leads to plastic shearing and work hardening on the surface of Ti6Al4V alloy. Generally, the development of cracks on the worn surface will then occur, consequently resulting in surface scraping and wear debris. When the wear debris and particles slide on the worn surface, plastic shearing occurs again, and tribo-chemical reactions may take place. Thus, the wear process may be accelerated along with the increase in the friction coefficient. Additionally, the transfer of worn materials will also occur during the sliding tribological test [40,41,42]. As illustrated in Figure 11, the USGP sample has the most significant area containing Fe content, while the polished sample contains the highest O content. There is no doubt that the O element comes from the oxidational wear and the Fe element comes from the worn material transfer of the GCr15 steel ball during the test. Mechanical alloying and frictional heating, which are attributed to a large amount of plastic deformation and wear debris sliding, are considered the reasons for oxidational wear and O element transfer [43]. According to Fe element analysis in Figure 11a,b, it is visible that Fe content significantly decreases when grooves exist on the surface of the Ti6Al4V alloy. Similar phenomena can be found in Figure 11c,d. Therefore, the grooves on the contact surface are beneficial to reduce worn material transfer and enhance the wear resistance of the Ti6Al4V alloy.

**Figure 12:**
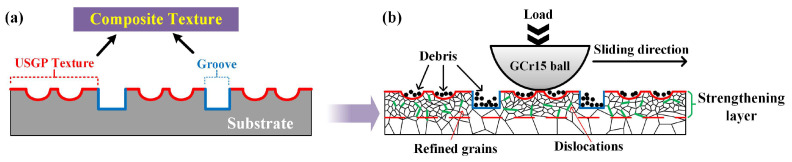
The sliding wear mechanism of the ULT sample: microstructure schematic diagram (**a**) and friction and wear mechanism model (**b**).

According to the above analysis, a composite texture, which consists of irregular micro-pits, granular bumps, and laser texture with molten pools, forms on the surface of the ULT sample. Thus, it can reduce the contact area, trap all kinds of wear debris, avoid three-body abrasive wear, and reduce the scratches of wear debris on the worn surface in the sliding process. As illustrated in Figure 12, after the USGP treatment followed by laser texturing, the contact load condition at the interface has changed, making the hard peaks of the micro-pits and the laser texture bear most of the load. This can alleviate the negative effect caused by the direct squeezing of wear particles and debris. Moreover, owing to the composite texture, a uniform gradient structure forms, and thus the stress concentration can be reduced effectively. Furthermore, a strengthening layer with high hardness, high dislocation density, and grain refinement was formed by USGP treatment. The high hardness and dislocation density can enhance the wear resistance properties of the contact surface, which is beneficial in reducing the adverse effects of wear particles and debris [44,45]. The surface hardness of all samples is not visible compared to the upper grinding piece of a GCr15 steel ball. Then, the peaks of the treated surface would be removed and tended to a similar roughness, easily, during the running-in stage of the tribological test. Hence, the surface roughness has little effect on the wear resistance. Finally, the essence of USGP treatment followed by laser texturing to improve the wear resistance of the contact surface was achieved by reducing the generation of wear abrasives. This practical method supports the possibility of effectively extending the wear resistance of the Ti6Al4V alloy.

## 4. Conclusions

In this study, the tribological properties of Ti6Al4V treated by USGP and laser texturing treatments were investigated. The results demonstrated that the lowest and most stable friction coefficients were obtained in the ULT sample with a hardened layer of 220 μm and a surface roughness of 1.42 μm, showing the best wear resistance. Compared to the polished, LT, and USGP samples, the friction coefficient of the ULT sample was remarkably decreased by 58%, 55%, and 39%, respectively. These results could be attributed to the high-velocity impact of the mixed abrasives on the machined surface during USGP treatment. The grain refinement and dislocation were formed on the treated surface layer. Finally, high hardness and strength were induced in the surface layer of Ti6Al4V, improving wear resistance properties. This work revealed that the USGP treatment followed by laser texturing is a promising method to overcome the poor wear resistance of Ti6Al4V alloy in engineering applications.

## Figures and Tables

**Table 1:** Chemical composition of Ti-6Al-4V titanium alloy (in weight, wt %).

Al	V	Fe	Si	Si	C	N	H	O	Ti
6.50	4.00	0.30	0.28	0.55	0.002	0.003	0.02	0.13	Bal.

**Table 2:** The technological parameters for laser texturing processing.

Processing Speed (mm/s)	Laser-Pulse Frequency (kHz)	Laser Focus Diameter (μm)	Wavelength (nm)	Output Power (w)
200	30	30	1053	10

## Data Availability

The data presented in this study are available in the article.
